# Emergence of a novel subpopulation of CC398 *Staphylococcus aureus* infecting animals is a serious hazard for humans

**DOI:** 10.3389/fmicb.2014.00652

**Published:** 2014-12-05

**Authors:** Nathalie L. van der Mee-Marquet, Anna Corvaglia, Marisa Haenni, Xavier Bertrand, Jean-Baptiste Franck, Jan Kluytmans, Myriam Girard, Roland Quentin, Patrice François

**Affiliations:** ^1^Service de Bactériologie et Hygiène, Centre Hospitalier Régional UniversitaireTours, France; ^2^UMR 1282, Infectiologie et Santé Publique, Université Francois RabelaisTours, France; ^3^Genomic Research Laboratory, University of Geneva HospitalsGeneva, Switzerland; ^4^Unité Antibiorésistance et Virulence Bactériennes, Agence Nationale de Sécurité Sanitaire (Anses)Lyon, France; ^5^Service d'Hygiène, Centre Hospitalier UniversitaireBesançon, France; ^6^UMR 6249 Chrono-environnement, Université de Franche-ComtéBesançon, France; ^7^Laboratory for Microbiology and Infection Control, Amphia Hospital, Breda and VU University Medical CenterAmsterdam, Netherlands

**Keywords:** bacteriophage, lysogeny, virulence factor, genome plasticity, genome content

## Abstract

Until recently, *Staphylococcus aureus* from clonal complex (CC)398 were mostly described as colonizing asymptomatic raised pigs and pig-farmers. Currently, the epidemiology of the CC398 lineage is becoming more complex. CC398 human-adapted isolates are increasingly being identified in bloodstream infections in humans living in animal-free environments. In addition, CC398 isolates are increasingly responsible for invasive infections in various animals. CC398 isolates that colonize asymptomatic pigs and the isolates that infect humans living in animal-free environments (human-adapted isolates) both lack several clinically important *S. aureus*–associated virulence factors but differ on the basis of their prophage content. Recent findings have provided insight into the influence of a φMR11-like helper prophage on the ability of CC398 isolates to infect humans. To assess the recent spread of the CC398 lineage to various animal species and to investigate the links between the φMR11-like prophage and the emergence of CC398 isolates infecting animals, we studied 277 isolates causing infections in unrelated animals. The prevalence of CC398 isolates increased significantly between 2007 and 2013 (*p* < 0.001); 31.8% of the animal isolates harbored the φMR11-like prophage. High-density DNA microarray experiments with 37 representative infected-animal isolates positive for φMR11-like DNA established that most infected-animal isolates carried many genetic elements related to antimicrobial resistance and virulence genes, and a φ3 prophage encoding immune-modulating proteins and associated with animal-to-human jumps. Our findings suggest recent clonal expansion and dissemination of a new subpopulation of CC398 isolates, responsible for invasive infections in various animals, with a considerable potential to colonize and infect humans, probably greater than that of human-adapted CC398 isolates, justifying active surveillance.

## Introduction

*Staphylococcus aureus* belonging to the clonal complex (CC) 398 has become a worldwide health threat (van Belkum et al., 2008). Until recently, CC398 isolates were mostly described as colonizing asymptomatic raised pigs and pig-farmers, and rarely infecting farmers and veterinarians (Huijsdens et al., 2006). Currently, the epidemiology of the CC398 lineage is becoming more complex. The lineage is now extending its territory as CC398 isolates are increasingly being identified in invasive infections in humans living in animal-free environments (Valentin-Domelier et al., 2011; Uhlemann et al., 2012; Verkade et al., 2012), in pet dogs, cats, and horses (Loncaric et al., 2014) and in animals of livestock species, including poultry, cattle, and rabbits (Argudín et al., 2013; Agnoletti et al., 2014).

CC398 isolates that colonize asymptomatic pigs (named below colonizing isolates) and the strains that infect humans living in animal-free environments (human-adapted isolates) both lack several clinically important *S. aureus*–associated virulence factors (Valentin-Domelier et al., 2011) but differ on the basis of their prophage content (McCarthy et al., 2011; Price et al., 2012). Phages play a critical role in bacterial diversity and evolution. They serve as a driving force in microbial evolution by transducing genes that supply their host with novel genetic information. This may enable host adaptation to new environments, and in some instances, confer virulence properties associated with pathogenesis in bacterial infections (Canchaya et al., 2004). Recent findings have provided insight into the influence of a helper prophage on the ability of CC398 isolates to infect humans. The sequence of this prophage named φMR11-like, shows high homology with that of φMR11, a phage isolated from a bacterium infecting the human bloodstream that is a candidate therapeutic phage (Matsuzaki et al., 2003). The φMR11-like prophage is specifically associated with the human-adapted CC398 isolates, and interacts with a human-associated β-converting φ3 prophage encoding immune-modulating proteins CHIP and SCIN such that virulence genes are expressed during stress situations and during lysogeny (van der Mee-Marquet et al., 2013).

The prophage content of CC398 isolates responsible for infections in animals has not been thoroughly investigated. Here, we report an analysis of a large collection of *S. aureus* isolates responsible for infections in various animal species. We determined the prevalence of CC398 isolates among these infected-animal isolates, and of isolates harboring the φMR11-like prophage which has been found to be associated with the human-adapted CC398 subpopulation. Then, using high-resolution whole-genome microarrays, we compared the mobile genetic element (MGE) content of infected-animal isolates harboring the φMR11-like prophage and of isolates belonging to the colonizing and human-adapted CC398 subpopulations.

## Materials and methods

### Bacterial isolates

We studied 292 isolates. The collection contains three subpopulations of isolates: (1) 277 epidemiologically independent isolates recovered from infected animals, between 2007 and 2013, in geographically distant locations in France (*n* = 263) and western Switzerland (*n* = 14); these isolates were obtained from 101 cows, 10 pigs, 15 rabbits, 29 chickens, 28 horses, 27 dogs, 20 cats, 40 sheep, three goats, and four primates; (2) eight colonizing CC398 isolates: four isolated from asymptomatic farmers and four from asymptomatic pigs; (3) four human-adapted CC398 isolates from epidemiologically unrelated French patients with bloodstream infection and not exposed to animals or farming (van der Mee-Marquet et al., 2013). The reference strains COL, Newman, and RF122 were also included for comparison purposes.

### Epidemiological study

We used the disk diffusion method (Bio-Rad, France) to test the antibiotic susceptibility of isolates. The mecA gene was detected by PCR. All isolates were tested by PCR to identify those that are CC398 isolates (Stegger et al., 2011); positive isolates were assigned to CC398 by MLST (Robinson and Enright, 2004). CC398 isolates were spa-typed using a previously published procedure (Frenay et al., 1996). We used a previously developed PCR-based test that targets three MR11-like prophage sequences (van der Mee-Marquet et al., 2013) to search for MR11-like prophage in the genome of the isolates.

### Analysis of MGE content by DNA microarray

A set of 37 isolates, representative of the diversity of the infected-animal isolates positive for φMR11-like DNA, was established for extensive MGE content analysis. The 37 isolates were chosen based on their antibiotic susceptibility profile, *spa*-type and host species origin. We used a microarray produced by *in situ* synthesis of 60 base-long oligonucleotide probes (Agilent, Palo Alto, CA), selected as described previously (Charbonnier et al., 2005). The array covers >96% of all open reading frames (ORFs), including those on plasmids, annotated in strains N315, Mu50, MW2, COL, NCTC8325, USA300, MRSA252, MSSA476, and Newman. Purified genomic DNA (gDNA) was extracted from each strain used for the design of the microarray (DNeasy; Qiagen), labeled with Cy5 dCTP with the Klenow fragment of DNA polymerase I (BioPrime, Invitrogen, Carlsbad, CA), and used for normalization (Talaat et al., 2002). Cy5-labeled pooled DNA (500 ng) and Cy3-labeled DNA from the test strain were mixed, diluted to 50 μl in Agilent hybridization buffer and hybridized with the microarray at a temperature of 60°C for 17 h in a dedicated hybridization oven (Robbins Scientific, Sunnyvale, CA). The slides were washed, dried under a nitrogen flow, and scanned (Agilent, Palo Alto, CA) with 100% photon multiplier tube power for both wavelengths. Fluorescence intensities were extracted with Feature Extraction software (version 9; Agilent). Local background-subtracted signals were corrected for unequal dye incorporation or unequal load of the labeled product. The algorithm consisted of a rank consistency filter and a curve fitted with the default LOWESS (locally weighted linear regression) method. Data were analyzed with GeneSpring, version 8.0 (Silicon Genetics, Redwood City, CA).

### Statistical data

Chi-squared tests and Fisher's exact test (two-tailed) were used to test for statistical significance; a *P* < 0.05 was considered significant.

### Ethics statement

#### Animal isolates

The 263 French isolates were collected from infected animals during the period 2007–2013 in diverse regions of the country via the Resapath network, which ensures the surveillance of antimicrobial resistance in diseased animals in France (www.resapath.anses.fr). Fourteen bovine isolates were recovered from cases of subclinical bovine mastitis in animals on Swiss farms. Isolates from Dutch pigs were obtained by non-invasive sampling during a field study. All studies were performed in accordance with the national guidelines and consequently did not require the approval of an ethics committee; the farmers gave informed consent and agreed to the collection of samples.

#### Human isolates

The French human isolates were obtained from clinical samples during annual surveillance studies that were run in accordance with the French Healthcare recommendations for the prevention of infection. Ethical approval of the monitoring programs was obtained from the appropriate national committee: the Réseau Alerte Investigation Surveillance des Infections Nosocomiales. The human isolates from the Netherlands were obtained during prospective surveillance, approved by the medical ethics committee of the Sint Elisabeth Hospitaal, Tilburg, the Netherlands (protocol number 0749). All participants provided written, informed consent.

## Results

### Epidemiological study

Sixty-nine of the 277 infected-animal isolates were identified as belonging to the CC398 lineage (24.9%) (Table 1). The prevalence of CC398 isolates was significantly higher during the period 2011–2013 (33.8%) than during the period 2007–2010 (3.6%) (*p* < 0.001). CC398 isolates were significantly more prevalent among isolates from rabbits and horses than those from the other host species (60.5% for rabbits and horses vs. 18.4% for the other species combined, *p* < 0.001).

**Table 1:** **Prevalence of CC398 isolates and of φMR11-like prophage elements in the genomes of the 277 animal isolates studied**.

**Host species**		**CC398 isolates (%)**	**Isolates positive for φMR11-like prophage (%)^a^**		
**All isolates**				**in CC398**	**in other lineages**
Cattle	101	20 (20)	15 (15)	9/20 (45)	6/81 (7)
Pig	10	3 (30)	2 (20)	0/3	2/7 (29)
Rabbit	15	12 (80)	12 (80)	12/12 (100)	0/3
Poultry	29	5 (17)	21 (72)	2/5 (40)	19/24 (79)
Horse	28	14 (50)	18 (64)	9/14 (64)	9/14 (64)
Dog	27	8 (30)	8 (30)	2/8 (25)	6/19 (32)
Cat	20	5 (25)	11 (55)	0/5	11/15 (73)
Primate	4	1 (25)	1 (25)	0/1	1/3 (33)
Sheep	40	0	0	–	0/40
Goat	3	1 (33)	0	0/1	0/2
All	277	69 (25)	88 (32)	34/69 (49)	54/208 (26)
**MRSA**					
Cattle	16 (16)	16/16 (100)	7/16 (44)	7/16 (44)	–
Pig	1 (10)	1/1	0/1	0/1	–
Rabbit	2 (13)	0/2	0/2	–	0/2
Poultry	1 (3)	1/1	0/1	0/1	–
Horse	25 (89)	14/25 (56)	15/25 (60)	9/14 (64)	6/11 (54)
Dog	18 (67)	5/18 (28)	6/18 (33)	1/5 (20)	5/13 (38)
Cat	20 (100)	5/20 (25)	11/20 (55)	0/5	11/15 (73)
Primate	1 (25)	1/1	0/1	0/1	–
Sheep	5 (12)	0/5	0/5	–	0/5
Goat^b^	0	–	–	–	–
All MRSA	89 (32)	43/89 (48)	39/89 (44)	17/43 (39)	22/46 (48)
**MSSA**					
Cattle	85 (84)	4/85 (5)	8/85 (9)	2/4 (50)	6/81 (7)
Pig	9 (90)	2/9 (22)	2/9 (22)	0/2	2/7 (29)
Rabbit	13 (87)	12/13 (92)	12/13 (92)	12/12 (100)	0/1
Poultry	28 (97)	4/28 (14)	21/28 (75)	2/4 (50)	19/24 (79)
Horse	3 (11)	0/3	3/3 (100)	–	3/3 (100)
Dog	9 (33)	3/9 (33)	2/9 (22)	1/3 (33)	1/6 (17)
Cat^b^	0	–	–	–	–
Primate	3 (75)	0/3	1/3 (33)	–	1/3 (33)
Sheep	30 (88)	0/35	0/35	–	0/35
Goat	3 (100)	1/33 (33)	0/3	0/1	0/2
All MSSA	188 (68)	26/188 (14)	49/188 (26)	17/26 (65)	32/162 (20)

a *isolates with at least one positive PCR test were considered positive*.

b *no isolate*.

Of the 277 infected-animal isolates, 89 were MRSA (32.1%), 106 were Tet^R^ (38.3%) and 55 were Ery^R^ (19.8%). The prevalence of resistance to methicillin was significantly higher among CC398 isolates than isolates of other lineages (62% among CC398 vs. 22.1% among non-CC398 isolates, *p* < 0.001). In the CC398 group, susceptibility to methicillin was significantly associated with isolates from infected rabbits (100% among rabbit CC398 vs. 24.6% among non-rabbit CC398 isolates, *p* < 0.001).

The *spa*-type t011 was the most common among the 69 CC398 isolates (44.9%), and there were eight other *spa*-types: t034, t571, t899, t1190, t108, t002, t097, and t2922. Four *spa*-types (t011, t034, t571, and t899) have previously been found in colonized-pig-associated CC398 isolates (1–3). The *spa*-type t1190 was only observed in isolates from rabbits, and t002 in isolates from poultry.

φMR11-like DNA was detected in 88 of the 277 isolates from infected animals (31.8%) and was significantly more frequent among strains isolated during the period 2011–2013 (40.5%) than during the period 2007–2010 (9.7%) (*p* < 0.001) (Table 1). The prevalence of φMR11-like prophage fragments was (i) higher among isolates from rabbits (80.0%), poultry (72.4%), and horses (64.3%) than other hosts, (ii) significantly higher among MRSA (43.8%) than MSSA (26.1%) isolates (*p* = 0.003), and (iii) significantly higher among CC398 isolates (49.3%) than isolates of other lineages (26.0%) (*p* < 0.001).

### DNA microarray analysis of the MGE content of genomes in isolates positive for ΦMR11-like DNA from infected animals, colonizing CC398 isolates, and human-adapted CC398 isolates

We studied 37 isolates (Table 2) representative of the 88 infected-animal isolates positive for φMR11-like DNA. On the microarrays, 351 probes appeared to be discriminative for the CC398 lineage (van der Mee-Marquet et al., 2013) (Supplementary Files 1–8). The 351 probes can be classified into four groups: 26 probes related to human-associated β-converting φ3-prophage (Supplementary File 1); 24 probes related to φMR11-like-prophage (Supplementary File 2); 38 φL54a-related probes (Supplementary File 3); and 263 probes related to diverse elements of the accessory genome of *S. aureus* (Supplementary Files 4–8). This last group includes 70 probes related to diverse prophages (Supplementary File 4), 39 MGE-related probes (Supplementary File 5), 18 probes related to antimicrobial resistance-associated elements (Supplementary File 6), 48 probes related to putative virulence-associated elements (Supplementary File 7), and 88 probes related to other diverse genetic elements (Supplementary File 8).

**Table 2:** **Characteristics of the 37 animal isolates studied with high resolution microarrays**.

**Host species**	**Year of isolation**	**Isolate**	**CC**	**Spa-type**	**Antibiotic resistance**		
					**Met^R^**	**Tet^R^**	**Ery^R^**
Cattle	2011	11329	398	t011	+	+	
	2012	32348	398	t899	+	+	
	2011	bovSW	398	t899	+	+	
	2011	32086	398	t011	+	+	+
	2011	11104	398	t011		+	+
	2010	M52	151	t529			
	2007	20263	new	t092			
	2013	33612	new	t529			
Pig	2013	34048	new	t1130		+	
	2012	34050	new	t318			
Rabbit	2013	34053	398	t1190		+	+
	2012	34066	398	t1190			
Poultry	2013	33972	398	t011		+	+
	2013	33924	398	t002		+	
	2012	33982	1495	t002		+	
	2013	33928	1495	t3478			
	2012	33983	1495	t002			
	2013	33963	new	t056		+	
Horse	2010	26023	398	t011	+	+	
	2013	33877	1	t1508			
	2012	32718	8	t394	+	+	+
	2012	32721	8	t394	+	+	+
Dog	2012	32978	398	t011	+	+	
	2013	k1343	398	t6605			
	2011	27741	8	t211	+		
	2011	27267	8	t2054	+		+
	2011	27095	8	t121	+		
	2012	32313	8	t008	+		
Cat	2012	32811	5	t002	+		
	2010	25175	5	t002	+	+	+
	2012	32820	5	t777	+		
	2012	32775	5	t003	+		+
	2010	26695	8	t574	+		+
	2010	26451	8	t068	+		+
	2012	32305	8	t008	+		
	2011	27744	22	t032	+		
Primate	2012	33318	new	t318		+	

**Figure 1:**
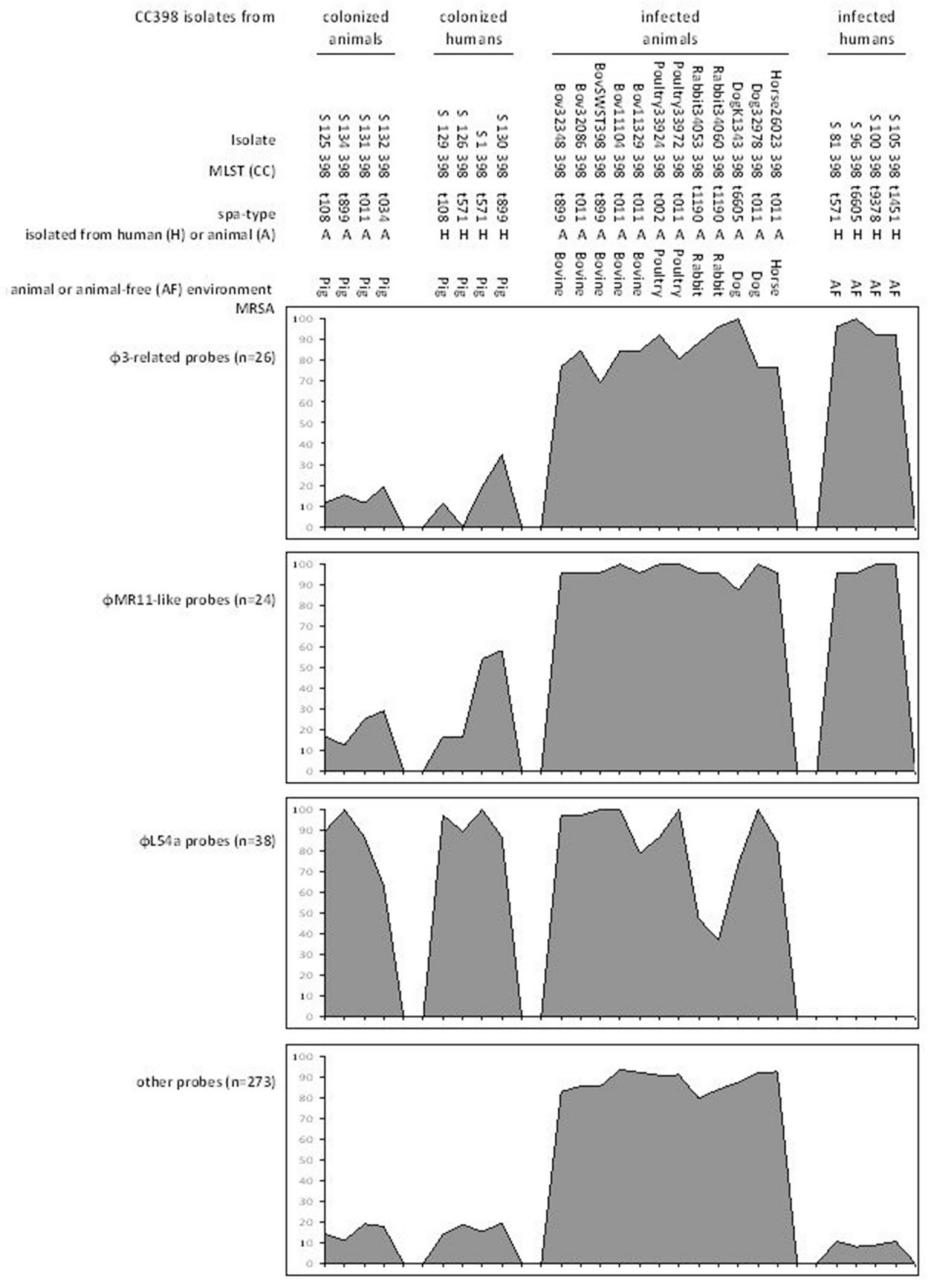
**Prevalence of hybridization of the four groups of probes with the genomes of the tested isolates belonging to CC398**. The different subpopulations of isolates showed different hybridization patterns. The colonizing isolates mostly only hybridized with probes for the φL54a prophage whereas the human-adapted isolates mostly only hybridized with the probes related to the φ3- and φMR11-like prophages. By contrast, the infected-animal CC398 isolates hybridized with the vast majority of the capture probes.

**Figure 2:**
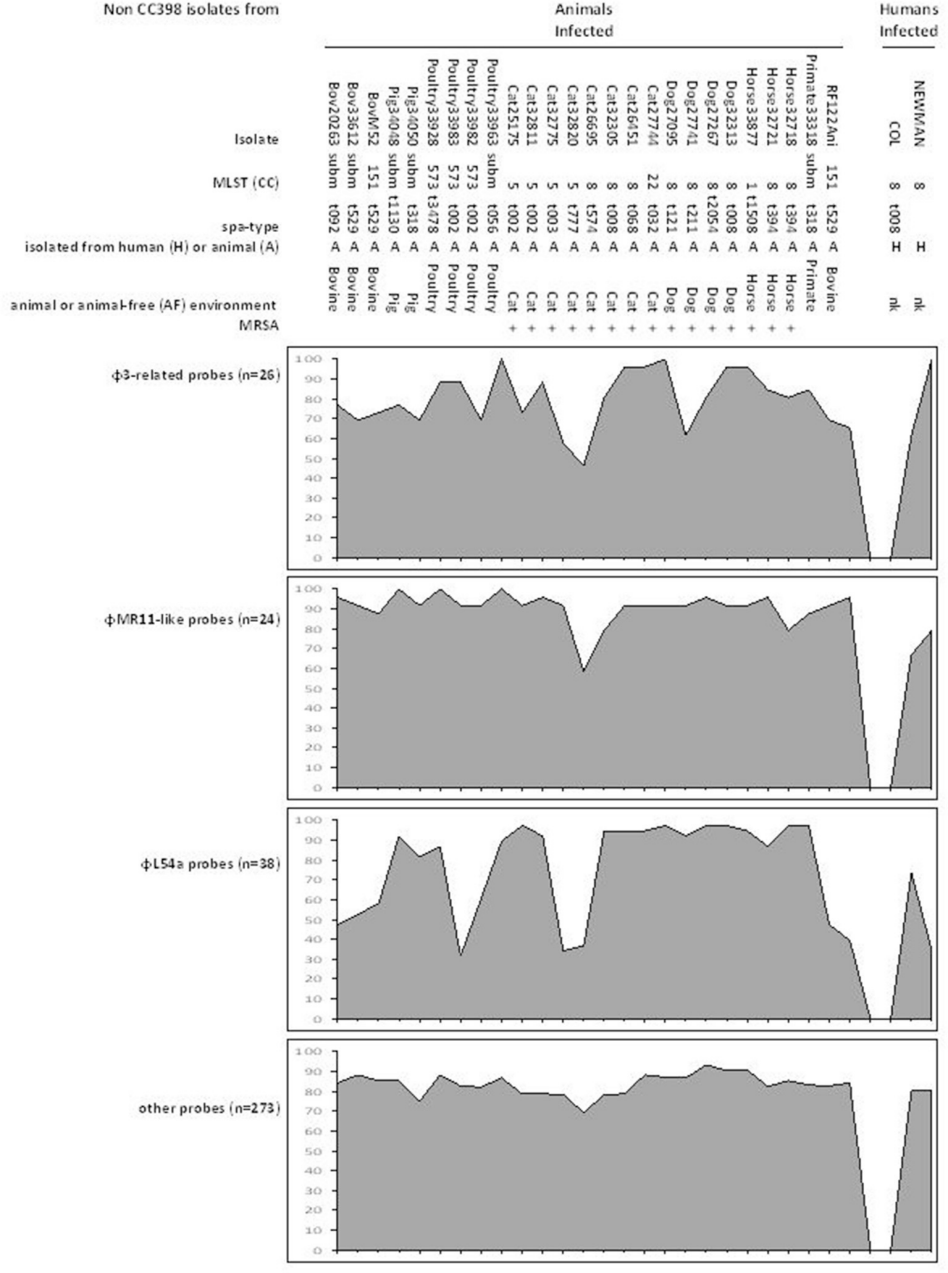
**Prevalence of hybridization of the four groups of probes with the genomes of the tested isolates belonging to other lineages**. The infected-animal isolates and the strains COL and NEWMAN responsible for invasive infections in humans hybridized with the vast majority of the capture probes.

Figures 1, 2 show the prevalence of hybridization signals for each isolate and each group of probes. The three different subpopulations of isolates (infected-animal, colonizing CC398, and human-adapted CC398) each showed different and characteristic hybridization patterns, and can thereby be clearly distinguished. Colonizing isolates and human-adapted isolates gave few positive hybridization signals. The colonizing isolates mostly only hybridized with probes for the φL54a prophage whereas the human-adapted isolates mostly only hybridized with the probes related to the φ3- and φMR11-like prophages. Few of the isolates in these two groups hybridized with probes of the large group of 263 probes related to diverse elements (Supplementary Files 4–8 and Figures 1, 2). By contrast, the infected-animal isolates hybridized with the vast majority of the capture probes recognizing the φ3 and φMR11-like prophages and numerous MGEs containing putative virulence genes and antibiotic resistance determinants. Most of the 37 infected-animal isolates, especially the MRSA isolates, also hybridized with the φL54a-related probes (Figures 1, 2). The infected-animal isolates all gave similar hybridization patterns, whether or not they belonged to the CC398, suggesting that the infected-animal isolates all possess similar set of accessory genes, regardless of their lineage of origin.

**Figure 3:**
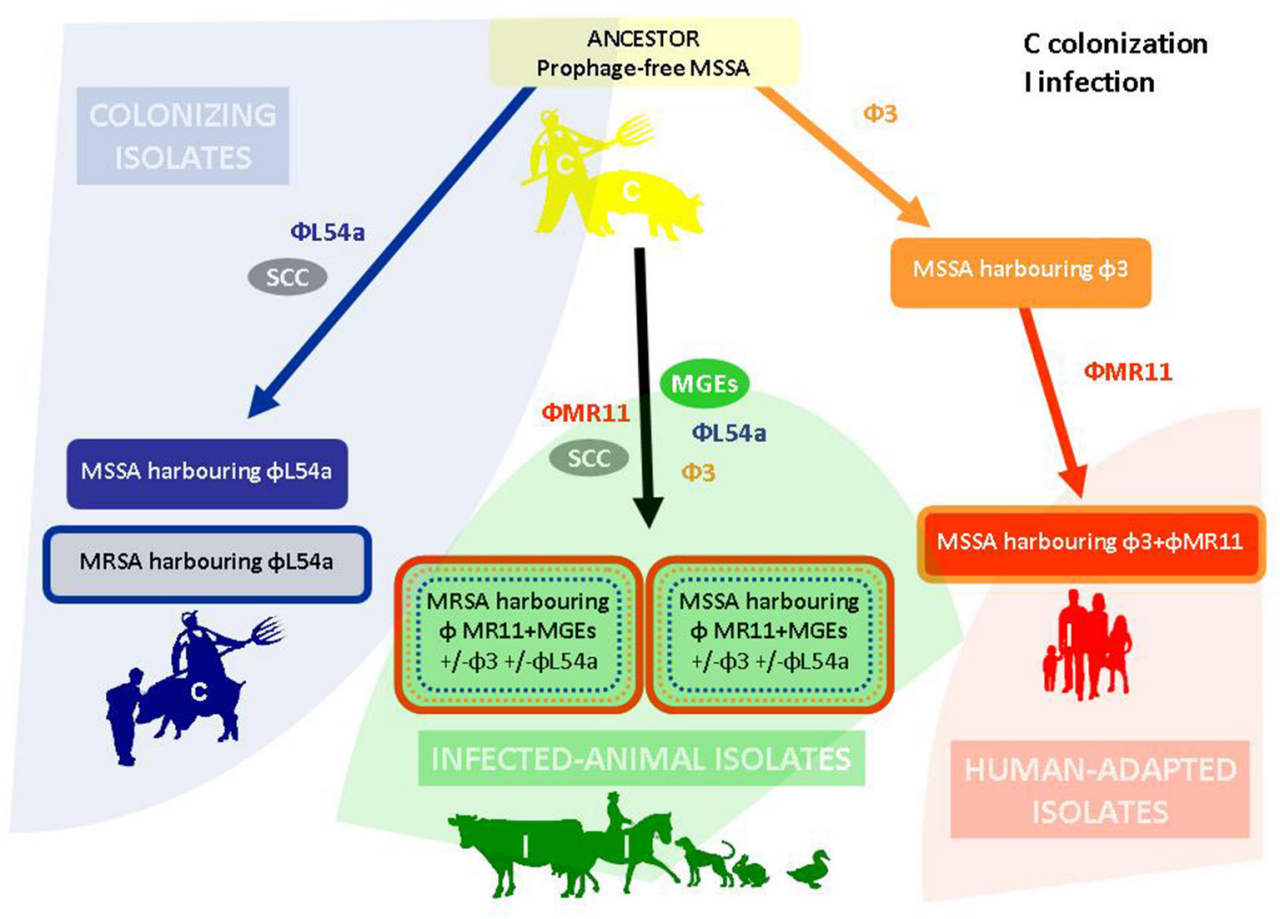
**Schematic representation of the diversification within the CC398 lineage**. Representatives of the ancestral prophage-free CC398 population were identified in the form of pig-borne colonizing isolates (upper part) (Hernandez et al., 2013; van der Mee-Marquet et al., 2013). The acquisition by such prophage-free isolates of the φ3 prophage and the φL54a prophage resulted in the ancestral MSSA isolates: human isolates carrying the φ3-prophage and pig-borne colonizing isolates carrying the φL54a prophage, respectively (Corvaglia et al., 2013; Hernandez et al., 2013; van der Mee-Marquet et al., 2013). The acquisition of the φMR11-like prophage by the human isolates resulted in human-adapted MSSA carrying φ3- and φMR11-like-prophages (Corvaglia et al., 2013; van der Mee-Marquet et al., 2013); the acquisition by the pig-borne colonizing isolates of the SCCmec cassette resulted in the pig-borne colonizing MRSA isolates (Hernandez et al., 2013; van der Mee-Marquet et al., 2013). The acquisition of MGEs, including the φMR11-like prophage, which contains genes contributing to bacterial virulence, resulted in the emergence of MSSA and MRSA isolates responsible for infections in both livestock and pet species (lower part).

Our analyses reveal genetic elements that the three CC398 subpopulations studied have acquired by horizontal genetic transfer. We propose a model in which the ancestral prophage-free colonizing CC398 MSSA isolates diverged into three subpopulations (Figure 3).

## Discussion

We studied a large set of *S. aureus* isolates representative of infections in animals. Our findings confirm that *S. aureus* CC398 isolates initially described as colonizers of asymptomatic pigs are now entering new territories, and infecting numerous animal species (van Belkum et al., 2008). Animal CC398 isolates, formerly exclusively associated with pigs and pig-farmers, have spread into other niches; and this event is evolutionarily recent. We show the chronology of this epidemiological evolution of the CC398 lineage: these isolates were able to infect only a limited range of hosts until 2007 but since 2010 have spread to numerous livestock and pet species. Note that our study only included a collection of selected strains and is therefore not representative of true incidence. Our findings need to be confirmed by, for example, prospective surveillance of the incidence of CC398 infections in these various animal species and in various geographical areas.

Population genetic studies have identified *S. aureus* clones with distinct genetic backgrounds as causative agents of infections in various host species. For example, sequence type (ST) 97 and ST151 appear to be ruminant-specific (Spoor et al., 2013) and ST5 is frequently isolated from poultry (Lowder et al., 2009), whereas ST5, ST8, ST22, ST30, and ST45 are mainly associated with human samples (Oliveira et al., 2002). By contrast, and consistent with recent reports (Argudín et al., 2013; Agnoletti et al., 2014; Loncaric et al., 2014), our work documents the capacity of strains from the CC398 lineage to colonize and adapt to numerous host species, including livestock, pets, and humans. For example, we show that a clone known to colonize pigs, MRSA-t011, is now frequently detected in cattle, poultry, horse, and dog. The CC398 isolates from infected animals nevertheless included two sub-clones that appeared host-specific: a rabbit-specific strain, MSSA-t1190 (Agnoletti et al., 2014) and a poultry-specific strain, showing the t002 *spa*-type usually detected in the avian-adapted ST5 lineage (Lowder et al., 2009). These observations suggest an appreciable genome plasticity of the CC398 lineage facilitating evolution leading to extended host diversity.

Our microarrays containing probes corresponding to numerous sequenced strains from diverse origins, cover a wide diversity of MGEs. Analyses with these microarrays indicated that among CC398 isolates, those colonizing asymptomatic pigs have the smallest pool of accessory genes. By contrast, infected-animal isolates had larger numbers of MGEs in their genomes. Many MGEs carry virulence genes and antimicrobial resistance determinants, consistent with our suggestion that these MGEs contribute to strain pathogenicity. These MGE were also observed in the genomes of strains COL and NEWMAN responsible for invasive infections in humans, and in the genome of strain RF122 responsible for infections in cows. This strongly suggests that these isolates have a considerable potential for virulence. In addition, the degree of conservation of MGEs among CC398 isolates suggests a recent clonal expansion and dissemination of this subpopulation of CC398 from infected animals.

Unlike colonizing isolates, both human-adapted and one third of the infected-animal CC398 isolates hybridized with φMR11-like related probes. As there is substantial similarity between the sequences of the φMR11-like prophage and Avβ, a prophage carrying avian niche-specific genes, our findings are consistent with evidence of an Avβ prophage in CC398 isolates from infected turkeys (Argudín et al., 2013). Thus, it appears that the φMR11-like prophage is associated with human-adapted CC398 isolates and also with isolates infecting various animal species. The carriage of a φMR11-like prophage was associated with the carriage of several SaPIs-like MGEs encoding putative virulence genes. The simultaneous presence of a helper phage and other MGEs, especially SaPIs, may result in significantly increased bacterial virulence (Ram et al., 2012). Therefore, our findings suggest that the interaction of the φMR11-like prophage and the MGEs encoding putative virulence genes may significantly alter bacterial pathogenicity. Using an animal model, we are currently investigating whether the φMR11-like prophage promotes virulence in CC398 infected-animal isolates.

There is evidence that acquisition of φ3 prophage facilitated animal-to-human jumps by CC97 isolates (Goerke et al., 2006; Spoor et al., 2013), leading to the spread of this pathogen. Most of the infected-animal isolates we studied carry a human-associated β-converting φ3 prophage encoding immune-modulating proteins. Therefore, it is likely that these CC398 infected-animal isolates are capable of adapting to, colonizing and finally infecting humans. Frequent contacts between humans and animals contribute to the transmission and spread of *S. aureus* between host species. Thus, our work suggests that livestock and pets colonized and/or infected with this third subpopulation of CC398 strains represent a major reservoir, favoring the emergence of CC398 *S. aureus* with the capacity for pandemic spread in humans.

## Conclusion

Our data support a model of rapid adaptation of the *S. aureus* CC398 lineage to diverse hosts. Originating from this lineage that formerly only colonized animals and humans in contact with them, two subpopulations emerged in parallel following the acquisition of MGEs. The common acquisition of the φMR11-like prophage by these two CC398 subpopulations coincide with epidemiological changes, particularly the evolution from colonizing hosts toward becoming agents responsible for severe invasive infections. Importantly, unlike human-adapted CC398 isolates that lack several clinically important *S. aureus*–associated virulence factors (Valentin-Domelier et al., 2011), the infected-animal isolates are similar to virulent *S. aureus* strains, harboring numerous genetic elements related to microbial resistance and virulence genes, and the β-converting φ3 prophage associated with animal-to-human jumps. This observation strongly suggests that these infected-animal isolates have a considerable potential for virulence, probably greater than that of human-adapted CC398 isolates. Given the specific features of the genomic content of the infected-animal CC398 isolates described here, our findings indicate that there is a need for prospective surveillance of both humans and animals to monitor the evolution of the CC398 lineage.

### Conflict of interest statement

The authors declare that the research was conducted in the absence of any commercial or financial relationships that could be construed as a potential conflict of interest.
