# Supplementary material for: Emergence of a novel subpopulation of CC398 Staphylococcus aureus infecting animals is a serious hazard for humans
Source: Front Microbiol. 2014 Dec 5;5:652. doi: 10.3389/fmicb.2014.00652 (PMC4257084; doi:10.3389/fmicb.2014.00652)
Supplement: Supplementary file 4 [file Table4.DOC]

**Supplementary file 4. Hybridization between additional phage-related probes and the genomes of the tested isolates. Black rectangles represent probes hybridizing to the corresponding genomes.**
